# Perspectives on Efforts to Address HIV/AIDS of Religious Clergy Serving African American and Hispanic Communities in Utah

**DOI:** 10.2174/1874613600701010001

**Published:** 2007-09-27

**Authors:** Stephen C Alder, Sara Ellis Simonsen, Megan Duncan, John Shaver, Jan DeWitt, Benjamin Crookston

**Affiliations:** University of Utah, Department of Family and Preventive Medicine, 375 Chipeta Way, Suite A, Salt Lake City, Utah 84105, USA

**Keywords:** HIV/AIDS, clergy, African American, Hispanic

## Abstract

**Introduction:**

The HIV/AIDS epidemic in America is rapidly progressing in certain subpopulations, including African-American and Hispanic communities. Churches may provide a means for reaching high-risk minority populations with effective HIV/AIDS prevention. We report on a series of focus group interviews conducted with Utah clergy who primarily serve African American and Hispanic congregations.

**Methods:**

A total of three focus groups (two with Catholic clergy serving Hispanic congregations and one with protestant clergy serving African American congregations) were conducted with eleven participants, lasting approximately two hours each. Each focus group was audio-recorded and transcribed for analysis. Analysis of the data was conducted using a modified grounded theory approach.

**Results:**

There were remarkable similarities in the attitudes and beliefs among all clergy participating in this study regarding HIV/AIDS and church-based prevention programs. All groups expressed concern about the diseases as a global epidemic and reported that the disease is highly preventable. Also, participants indicated a sense of responsibility to address the issues surrounding HIV/AIDS-related prevention, testing and care within their theological framework.

**Conclusion:**

HIV/AIDS prevention and care for the infected are seen as falling within the scope of religious organizations. Openness to expanding efforts in this regard was shared by clergy participating in this study. Approaching religious leaders with tailored approaches that respect the values and practices of their particular religions will be more effective than attempting to impose approaches that do not achieve this standard.

## INTRODUCTION

After two decades of HIV/AIDS in America, the epidemic is rapidly progressing in certain subpopulations, including African-American and Hispanic communities. Adults and children in these communities have been disproportionately affected. According to the CDC surveillance reports for 2001, African-Americans and Hispanics together represent 57 percent of reported AIDS cases and 62 percent of reported HIV cases [1]. In addition, both African American and Hispanic individuals infected with HIV/AIDS have poorer survival than infected Caucasian individuals [2]. Novel methods of reaching high risk minority communities are needed in order to decrease the transmission of HIV in these communities, increase the number of HIV-infected people who know their status, and increase the number of people who are linked to prevention and treatment services.

Churches may provide a means for reaching high-risk minority populations with effective HIV/AIDS prevention messages and provide a link to HIV-related testing and treatment services in a culturally acceptable manner [3]. The role of churches as focal points for spiritual, social, political and health related activities within minority communities has been previously described [4,5]. Religious leaders are viewed as credible sources of information and guidance, and frequently have contact with hard-to-reach populations [6]. In one study of individuals at high risk for HIV/AIDS, including many who were African American or Hispanic, 75 percent reported that religion was “somewhat” to “extremely” important and 60 percent reported having attended a church service at least once in the past three months [7].

Nationally, African American Christian churches have been among the first to incorporate HIV/AIDS programs into their ministry activities. However, even among African American churches, the extent of HIV/AIDS prevention messages reaching the congregation may be limited. These limitations have been further exacerbated by past failures to recognize the growing magnitude of the HIV/AIDS pandemic. For example, a 1994 survey of 176 African-American churches in the northeastern United States revealed that only 15 percent had youth sexuality programs, 15 percent had youth substance abuse programs and 3 percent had AIDS support programs [8]. In addition, a study of African-American Baptist ministers revealed that 37 percent believed HIV/AIDS posed “little risk” to their communities [9]. Churches with predominantly Hispanic congregations have been less likely to implement HIV/AIDS programs. Focus group data on HIV/AIDS education in Catholic schools and churches revealed that the topic of HIV/AIDS was often avoided or only mentioned briefly because of barriers such as concerns about dealing with the topic of homosexuality, denial of the risks associated with HIV/AIDS, and inexperience in discussing human sexuality [10].

Data providing insight into the role of churches relative to HIV/AIDS prevention activities, attitudes and beliefs among clergy, and barriers/facilitators to implementing programs are scarce. However, it appears that churches and religious organizations are underutilized resources for the dissemination of HIV/AIDS prevention messages. This may be due, in part, to sexual and illicit drug use topics that are central to HIV/AIDS prevention. Insight into the factors related to implementing church-based HIV/AIDS prevention programs may allow for much greater participation by churches in these activities. In addition, understanding theological issues that impact the willingness of church clergy to engage in HIV/AIDS prevention may provide insights into creative ways for impacting hard to reach, high risk populations. We report on a series of focus group interviews conducted with Utah religious leaders who primarily serve African American and Hispanic congregations.

## METHODS

This study was approved by the University of Utah Institutional Review Board. Study participants were identified by the Utah Catholic Diocese website in conjunction with the Utah Catholic Hispanic Ministry, the Utah Office of Minority Affairs and the Utah African American Community Resource Directory. Potential participants were sent a letter of invitation followed by a telephone call for those that did not respond to the initial request. Focus groups were held at either church properties or a public library.

Following study consent, clergy participated in a focus group interview. A total of 3 focus groups (2 with Catholic clergy serving Hispanic congregations and 1 with protestant clergy serving African American congregations) were conducted with eleven participants, lasting approximately 2 hours each.

The focus groups were conducted in the manner outlined by Krueger [11]. Focus group discussions were led by a trained moderator who utilized a set of semi-structured questions with field notes recorded by a separate member of the research team. Each focus group was audio-recorded and transcribed for analysis. Accuracy of transcription was checked by a separate member of the research team.

## DATA ANALYSIS

Analysis of the data was conducted using a modified grounded theory approach [12]. In traditional grounded theory analysis, data collection and analysis are concurrent. Because of the nature of the focus groups, data analysis began while focus groups were still being conducted. Two researchers conducted independent analysis of the data and compared their results. Further refining of categories was undertaken, and analysis was considered complete when both researchers felt that a satisfactory level of clarity and explanation had been reached.

## RESULTS

There were remarkable similarities in the attitudes and beliefs of Catholic and African-American clergy participating in this study regarding HIV/AIDS and church-based prevention programs. When asked about HIV/AIDS in general, both groups expressed concern about the diseases as a global epidemic and also stated that the disease is highly preventable. As well, all participants indicated a sense of responsibility to address the issues surrounding HIV/AIDS-related prevention, testing and care within their theological framework and spheres of influence.

### Role of Churches Relative to HIV/AIDS

When asked who is responsible for addressing HIV/AIDS within minority communities, the participants generally felt there were several aspects of HIV/AIDS that needed to be addressed by different groups. One respondent said, “…I would feel responsible on my level …medical professions would have to be responsible in a different way,… the educational community would have to feel responsibility in terms of getting the word out and devising ways of making the word important and effective.”

Participants discussed the importance of having a “moral” lifestyle and viewed their role as one of imparting moral values as a way of preventing HIV/AIDS. According to one respondent, “…moral orientation comes from the parents…and the communal aspects of the church are evolved in the handing down of our moral traditions … there’s really a two fold approach, from home, and reinforced by teachings of the church.”

Educating individuals about HIV/AIDS and helping them understand the consequences of risky behavior was also mentioned. Participants expressed discomfort with discussing HIV/AIDS prevention methods such as condom use from the pulpit. They communicated willingness to talk about lifestyle as a moral issue, but stated that they were uncomfortable discussing during religious sermons the worldly consequences of “immoral behavior” such as unwanted pregnancy and HIV infection. One minister stated, “Abstinence…is always 100 percent effective.”

Both the Catholic and the African American clergy discussed lifestyle issues such as sexual abstinence before marriage and complete fidelity within marriage, and stated that they encourage church members to uphold such standards. Both groups of clergy indicated belief that their promotion of moral lifestyles was directly addressing the HIV/AIDS epidemic.

### Impact of HIV/AIDS Prevention Efforts

The Catholic clergy indicated that HIV/AIDS does not have a large impact on members of their congregations. One Catholic priest stated, “…I haven’t run across a single person who has come to me with [HIV/AIDS] as a problem.” However, the African American clergy stated that they knew of church members who were infected as well as members who had died from the disease. As such, African American clergy indicated more awareness and active engagement in addressing HIV/AIDS within their congregations.

Both groups stated that members of their congregation engage in risky behaviors. They also discussed their belief that HIV/AIDS has a bigger impact on people outside of their congregation than on participating church members, and stated that those whose lifestyles put them at risk for HIV/AIDS may not attend or may feel ostracized from the church. Thus, they further indicated that outreach beyond their own church members was both necessary and part of their ministry. Included in this was a concern regarding HIV/AIDS among incarcerated individuals.

### Current Church-Based Activities addressing HIV/AIDS

All respondents professed compassion for those infected with HIV/AIDS and for those affected because of an HIV positive loved one. There was a distinction between teachings that condemn the behaviors and compassion for individuals engaged in these behaviors. When asked about current HIV/AIDS-related activities, the African American clergy discussed a number of established programs within their churches including HIV/AIDS information/awareness meetings, educational programs focused on youth, spirituality programs for those infected with HIV/AIDS, and outreach to prisoners and community members. The Catholic clergy discussed moral teachings targeted at high risk behaviors for youth prior to their Quinsañeras celebrations.

### Acceptable Church-Based HIV/AIDS Related Activities

Respondents were asked about the acceptability of a number of church-based HIV/AIDS prevention programs, and gave very similar answers. Both the Catholic and African American Christian clergy felt that sermons and youth services about abstinence and drug abuse, public workshops about HIV/AIDS prevention, support groups for people with HIV/AIDS and their family members, and outreach programs directed at people at risk for HIV/AIDS would be acceptable church-based activities. A number of participants stated that they would not discuss condom use or other non-abstinence-related prevention methods from the pulpit. Both groups felt that sermons and youth services about condom use, clean needle exchanges, and condom distribution would be unacceptable church-based activities.

Some participants stated that discussion of such issues may arise during one-on-one interactions with church members or in church-sponsored activities outside of sermons. In each case, such comments were linked to concerns about balancing appropriate moral teaching with effective means of preventing infections. They were clear that such discussions needed to be handled with the utmost sensitivity and judgment.

### Resources Needed by Churches for Effective HIV/AIDS Approaches

Ministers were eager to learn about means of improving their existing programs. Specifically, they were interested in learning more factual information about HIV/AIDS and developing programs that fit within their respective doctrines. They further discussed the need to be sensitive to doctrinal limitations, time constraints, and busy schedules in improving upon their current efforts. All were open to integrating their efforts with others addressing this epidemic as long as these same sensitivities were considered.

## DISCUSSION

With the continued emergence of HIV infections there is increasing evidence that preventive efforts are not sufficiently effective and that increased attention is needed to care for the infected. When addressing these issues, novel approaches such as church-based prevention programs may provide the means for increased success in both prevention and care. Our research indicates that religious leaders see themselves as having responsibility in addressing the HIV/AIDS epidemic. Further, they see themselves as a critical part of the multifaceted campaign to address HIV/AIDS related impacts.

Specifically, clergy indicated that there are many ways in which their efforts related HIV/AIDS may be enhanced. From their perspective, collaboration with others that fit within the scope and boundaries of their ministries will likely lead to increased effectiveness in prevention and compassionate care. Further attention is needed for the development approaches to enhance clergy members’ capacity to be effective in their efforts as well as the design of evidence-based interventions that are consistent with clergy members’ primary ministerial responsibilities.

Church-based programs may also be a means of reaching high-risk minority populations. Many outreach efforts are already targeting individuals at high risk, providing a natural platform from which to further extend HIV/AIDS related efforts. However, in the development of such programs, sensitivity to church doctrine, theological limitations, and cultural diversity are factors that must be addressed. With such consideration, HIV/AIDS prevention messages and promoting care for the infected can easily be incorporated into current church teachings and outreach programs.

Academic and other social institutions have a role in working with religious organizations to integrate efforts as a means of creating synergy that can directly impact the HIV/AIDS epidemic and those infected. Specific programs can be designed and collaboratively implemented that combine the most current understanding of the HIV/AIDS biology and effective behavioral and therapeutic interventions that are within the scope of churches stewardships. We found that not only were the clergy of churches serving minorities open to such collaboration, they were anxious for partnerships that respected their theological boundaries. Rather than attempting to impose approaches that are contrary to church values, interventions need further development that will allow for enhancing the impact of efforts that are deemed to be appropriate.

The primary limitations of this study include being geographically constrained and having a relatively small sample that may lack generalizeability. However, the primary intent of this study was an initial effort to explore the openness of religious clergy to engage in collaborative efforts related to HIV/AIDS. This work merely provides a glimpse of the potential to combine the influence of religious organizations with evidence-based approaches typically reserved for non-religious efforts targeting HIV/AIDS.

## CONCLUSION

HIV/AIDS prevention and care for the infected are seen as falling within the scope of religious organizations. Further, openness to expanding efforts in this regard was shared by clergy participating in this study. Critical next steps include more research to better understand the perspective of religious organizations and development of approaches that can be implemented within the context of their philosophical frameworks. Approaching religious leaders with tailored approaches that respect the values and practices of their particular religions will be more effective than attempting to impose approaches that do not achieve this standard. There was clear consensus among respondents that supportive partnerships rather than directive efforts are desired for increasing the effectiveness of HIV/AIDS related efforts.
